# CT and radiographic analysis of sagittal profile changes following thoracoscopic anterior scoliosis surgery

**DOI:** 10.1186/1748-7161-7-15

**Published:** 2012-08-22

**Authors:** Maree T Izatt, Clayton J Adam, Eugene J Verzin, Robert D Labrom, Geoffrey N Askin

**Affiliations:** 1 Paediatric Spine Research Group, Queensland University of Technology and Mater Health Services Brisbane Ltd, Queensland, Australia

**Keywords:** Thoracoscopic anterior spinal fusion, Anterior spinal fusion, Adolescent idiopathic scoliosis, Sagittal profile, Computed tomography (CT), Thoracic kyphosis, Lumbar lordosis

## Abstract

**Background:**

Previous studies report an increase in thoracic kyphosis after anterior approaches and a flattening of sagittal contours following posterior approaches. Difficulties with measuring sagittal parameters on radiographs are avoided with reformatted sagittal CT reconstructions due to the superior endplate clarity afforded by this imaging modality.

**Methods:**

A prospective study of 30 Lenke 1 adolescent idiopathic scoliosis (AIS) patients receiving selective thoracoscopic anterior spinal fusion (TASF) was performed. Participants had ethically approved low dose CT scans at minimum 24 months after surgery in addition to their standard care following surgery. The change in sagittal contours on supine CT was compared to standing radiographic measurements of the same patients and with previous studies. Inter-observer variability was assessed as well as whether hypokyphotic and normokyphotic patient groups responded differently to the thoracoscopic anterior approach.

**Results:**

Mean T5-12 kyphosis Cobb angle increased by 11.8 degrees and lumbar lordosis increased by 5.9 degrees on standing radiographs two years after surgery. By comparison, CT measurements of kyphosis and lordosis increased by 12.3 degrees and 7.0 degrees respectively. 95% confidence intervals for inter-observer variability of sagittal contour measurements on supine CT ranged between 5-8 degrees. TASF had a slightly greater corrective effect on patients who were hypokyphotic before surgery compared with those who were normokyphotic.

**Conclusions:**

Restoration of sagittal profile is an important goal of scoliosis surgery, but reliable measurement with radiographs suffers from poor endplate clarity. TASF significantly improves thoracic kyphosis and lumbar lordosis while preserving proximal and distal junctional alignment in thoracic AIS patients. Supine CT allows greater endplate clarity for sagittal Cobb measurements and linear relationships were found between supine CT and standing radiographic measurements. In this study, improvements in sagittal kyphosis and lordosis following surgery were in agreement with prior anterior surgery studies, and add to the current evidence suggesting that anterior correction is more capable than posterior approaches of addressing the sagittal component of both the instrumented and adjacent non instrumented segments following surgical correction of progressive Lenke 1 idiopathic scoliosis.

## Introduction

Surgical management of Adolescent Idiopathic Scoliosis (AIS) via the anterior approach has been shown to preserve motion segments, while producing major and compensatory curve corrections comparable to posterior approaches [1-9]. However, patients with AIS also exhibit a reduced thoracic kyphosis or hypokyphosis [10-13] accompanying the coronal and rotary distortion components. As a result, surgical restoration of the thoracic kyphosis while maintaining lumbar lordosis and overall sagittal balance is a critical aspect of achieving good clinical outcomes in AIS patients [5,14-16].

Anterior surgical approaches appear to be advantageous in this respect, with consistent reporting of increased thoracic kyphosis after surgery [4,5,7,8,14,16-19]. By contrast, previous literature has demonstrated flattening of thoracic sagittal contour and a corresponding decrease in lumbar lordosis following posterior pedicle screw instrumentation in the thoracic spine [7,16,20-22]. Furthermore, the occurrence of proximal junctional kyphosis following posterior stabilisation is high, with reported incidence between 9.2% and 46% [23-26].

When performing anterior approaches, thoracoscopic (keyhole) anterior spinal fusion (TASF) is an accepted alternative to open surgery in the instrumented correction of major thoracic curves [8,27-30]. The thoracoscopic approach reduces chest wall disruption, with less blood loss and soft tissue dissection than open procedures [4,8,31], and pulmonary function has been reported to recover as early as 12 months after surgery [32,33]. To the best of our knowledge, sagittal profile changes have been reported for four existing single centre cohorts of TASF patients to date [4,8,18,29,34-36], with reported increases of between 4 to 12° in thoracic kyphosis and 4 to 7° in lumbar lordosis on radiographs at minimum two years after TASF. Taken together, these studies suggest that TASF improves both thoracic kyphosis and lumbar lordosis in AIS patients.

**Figure 1:**
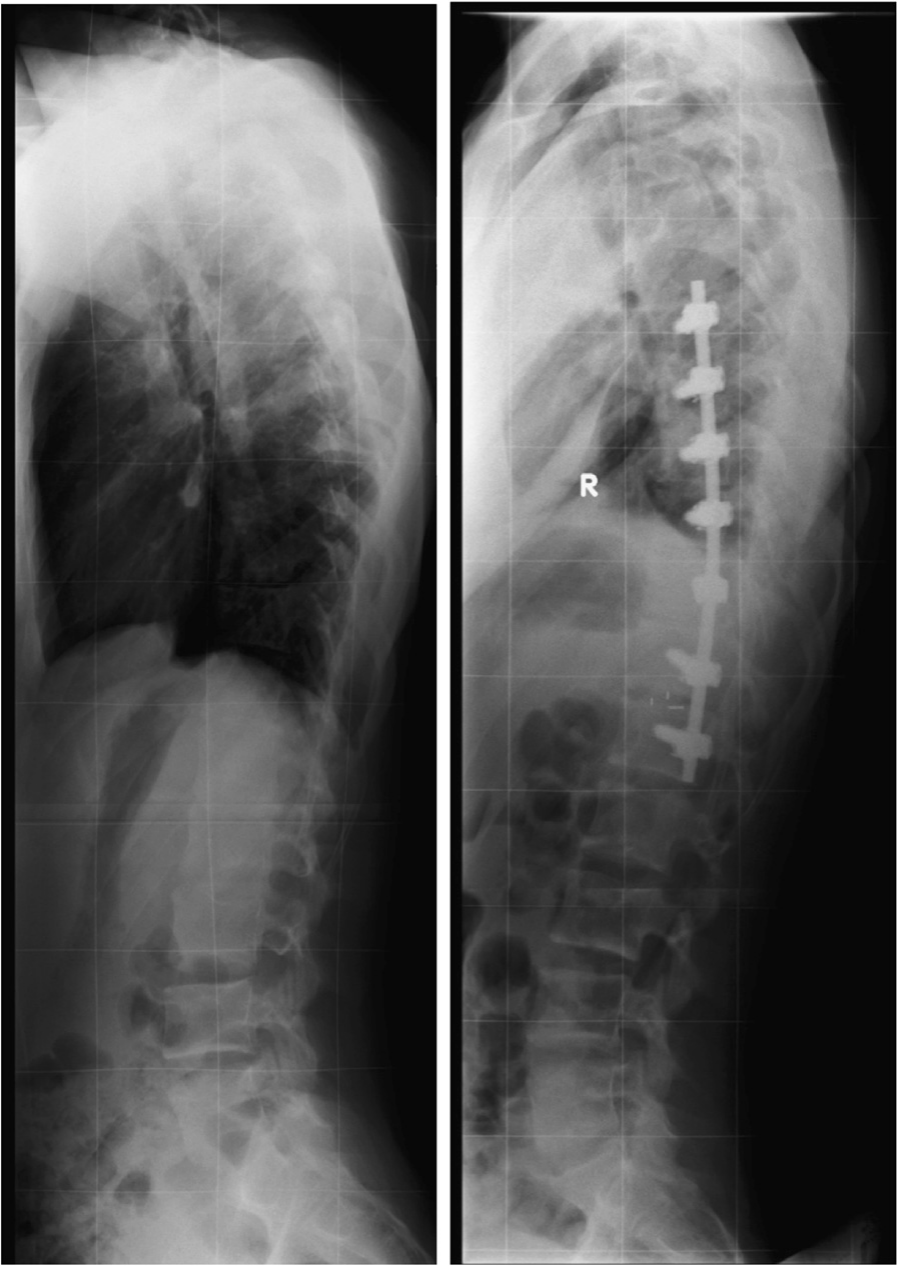
**Typical standing sagittal radiographs of an AIS patient before and after thoracoscopic anterior spinal fusion surgery illustrating the poor definition of vertebral endplates on radiographs**.

However, the existing studies raise several questions relating to sagittal profile changes following AIS surgery. Firstly, standing sagittal radiographs are notoriously poor quality (Figure 1) when attempting to identify thoracic endplates for sagittal Cobb angle measurement, adversely affecting measurement reliability [37-39]. Secondly, there has been a lack of reporting of localised sagittal profile measures such as the instrumented levels or the thoracolumbar junction. A third question is whether TASF affects the sagittal profile of hypokyphotic patients differently to normokyphotic patients. 

Accordingly, the aims of this study were to; (i) use the superior endplate clarity provided by low dose computed tomography (CT) scans to perform both overall and localised sagittal profile measurements before and after TASF for a group of AIS patients, (ii) compare these low dose CT sagittal profile measurements with standing radiograph sagittal profile measurements for the same patients, (iii) document the inter-observer variability associated with supine CT sagittal profile measurement, (iv) assess whether TASF affects hypokyphotic patients differently to normokyphotic patients, and (v) provide a quantitative comparison of previously reported sagittal profile results after posterior, open anterior and thoracoscopic anterior scoliosis correction literature to date.

## Materials and method

### Study cohort

Between November 2002 and January 2008 a subset of 30 patients from a large, single centre consecutive series of 198 patients who had undergone thoracoscopic anterior instrumented thoracic fusion were consented to participate. The surgeries were all performed by the two senior authors (GNA and RDL) at the Mater Children’s Hospital, Brisbane, Australia. The indication for surgery was progressive thoracic AIS classified as Lenke Type 1 with a Cobb angle ≥ 40° and a T5- T12 kyphosis Cobb angle ≤ 40°.

The study was performed prospectively after obtaining ethics committee approval from our institution to perform postoperative low dose CT scans at approximately two years after surgery on a subset of patients receiving thoracoscopic anterior scoliosis correction. The study data was gathered prospectively for all cases, from a larger case series. All patients scheduled for thoracoscopic surgery and all those a minimum of 24 months after surgery during the three year ethics approval period were invited to participate. The data obtained from this group of patients who had CT scans after surgery was collected to contribute to multiple clinical and biomechanical studies of anterior scoliosis correction surgery, of which this study is one aspect.

### Surgical technique

The surgical procedure was based on the technique first described by Picetti *et al*[40] and has been reported previously [33,41]. In all cases Endolegacy (Medtronic-Sofamor-Danek, Memphis, TN, USA) 4.5 mm (first 14 cases) or 5.5 mm (subsequent 16 cases) titanium spinal implants were used and intersegmental compression was applied across the rod to achieve curve correction. Rib head autograft was used in the initial five patients, and in all subsequent patients irradiated mulched allograft (Queensland Bone Bank) was used to facilitate interbody fusion which is now the standard practice. Levels for instrumentation were selected to include the end vertebrae of the major scoliotic curve. If instrumentation extended beyond T12, an interbody spacer cage was placed between T12-L1 to assist the spine’s transition into lordosis. Radio-translucent markers within the cage are visible on the postoperative radiograph (Figure 1).

### CT and radiographic evaluation

Postero-anterior (PA), sagittal and bending radiographs of the spine were obtained before surgery, as well as a clinically indicated thoracolumbar CT scan using a low-dose scanning protocol. A single CT scan before surgery was part of our surgical planning process at the time of this study for patients undergoing TASF to facilitate safer screw sizing and positioning [42]. At the two year review after surgery, the subset of patients enrolled in the study had the ethically approved low dose thoracolumbar CT scan for research purposes, in addition to the standard radiographs (PA and sagittal) to assess deformity correction.

Four different CT scanners were used over the six year period of the study; (i) a 4-slice Toshiba Aquilion (Toshiba Medical Systems, Tokyo, Japan) (ii) a 64-slice Philips Brilliance (Philips Healthcare, Andover, USA) (iii) a 64 slice GE Lightspeed Plus (GE Healthcare, Chalfont St. Giles, UK) and (iv) a 64 slice GE Lightspeed VCT (GE Healthcare, Chalfont St. Giles, UK). The scan coverage in each case was from C7 to S1. Dose reports were commissioned for all four scanners, and the highest estimated radiation dose of 3.7 mSv occurred with the oldest scanner (Toshiba Aquilion), with uncertainties due to the dose model in the order of ±20% [43]. By comparison, the combined dose for PA and sagittal standing radiographs is in the order of 1.0 mSv, and the annual background radiation in Queensland, Australia is approximately 2.0 mSv per annum. Estimated doses for the newer 64 slice scanners were substantially lower (in the order of 2 mSv).

**Figure 2:**
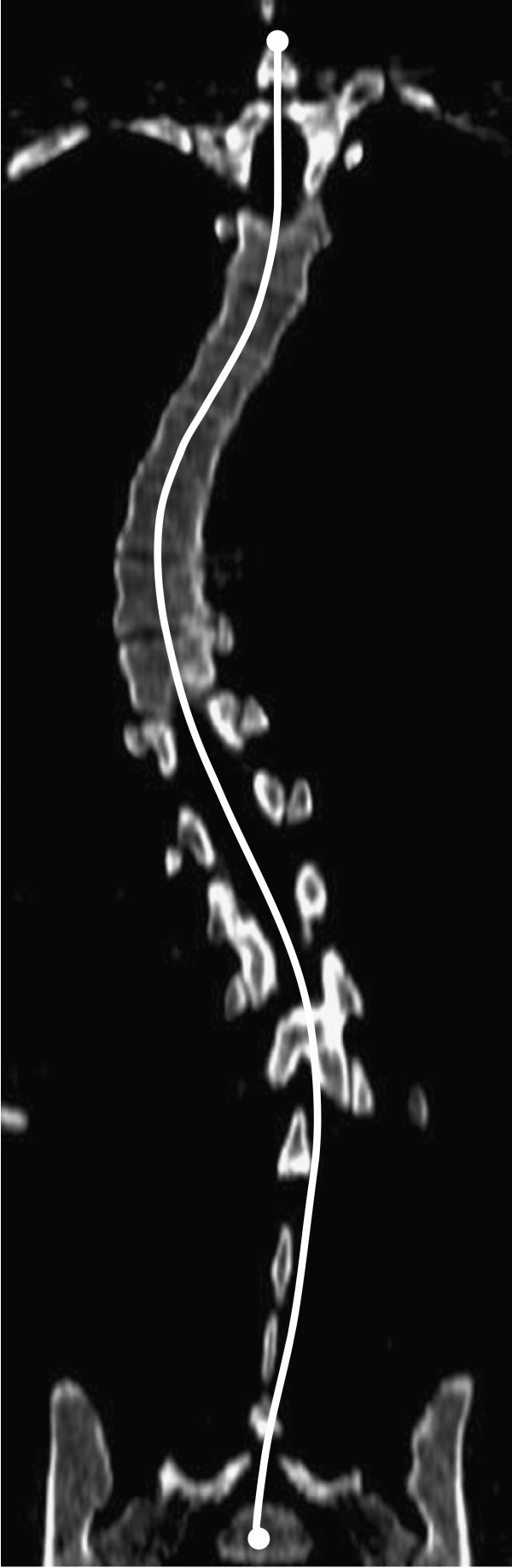
**Method of reformatting CT scan dataset to obtain sagittal section by tracing the path of the mid-sagittal plane to account for the scoliotic curvature in the coronal plane**.

**Figure 3:**
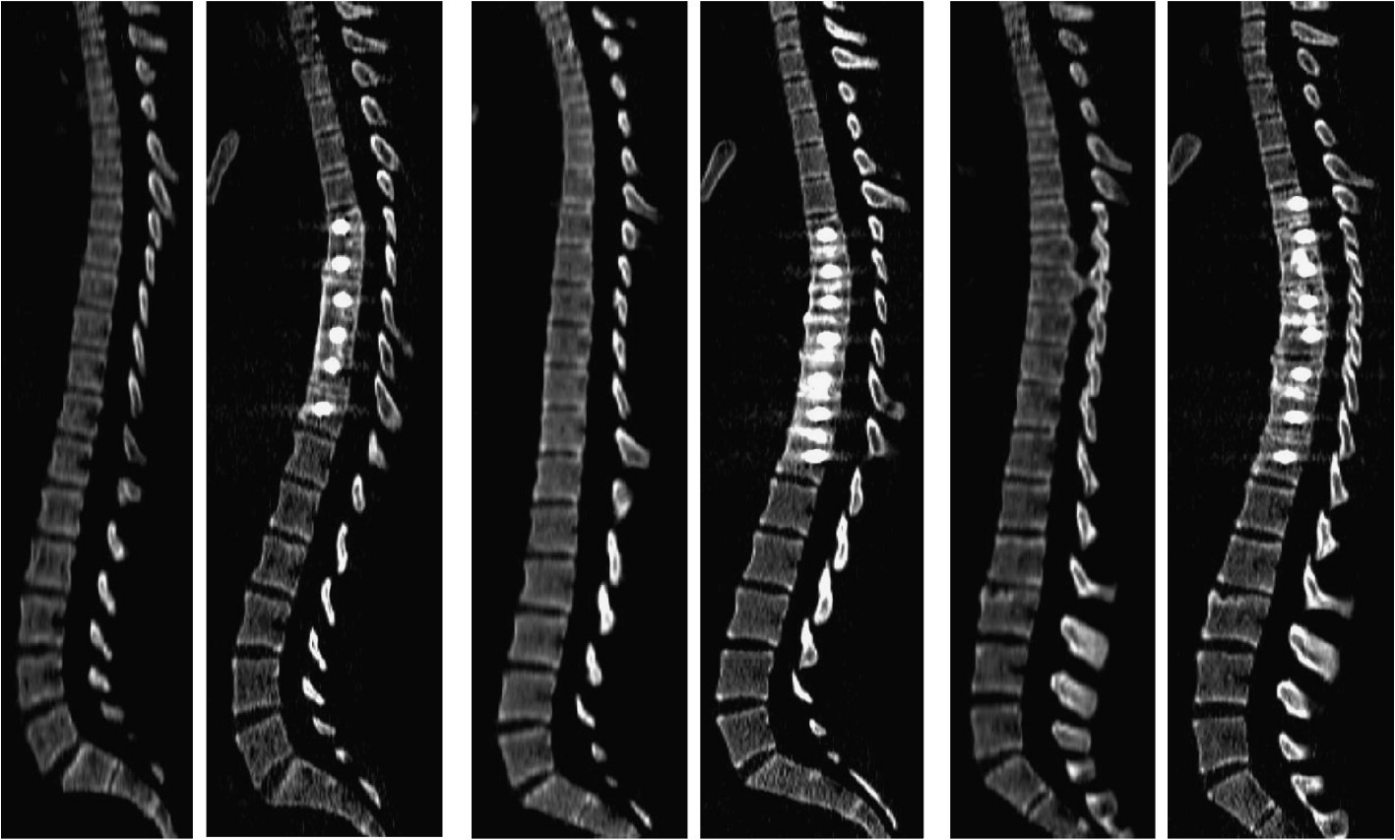
**Resulting reformatted sagittal images for three patients in the study showing the paired sagittal CT reconstructions before and after surgery for each patient.** Note the superior endplate definition compared to standing radiographs.

The ImageJ software (v. 1.42q, National Institutes of Health, USA) was used to create reformatted sagittal plane images from the transverse slices, analyse the scans and measure the desired vertebral endplate angles. Reformatting is required due to the lateral deviation of the spine as a result of the scoliosis deformity, which normally precludes the entire spine being visible on a single sagittal image. By firstly tracing the coronal plane deformity in ImageJ (Figure 2), the program is able to reformat the sagittal images into a single plane for analysis. Figure 3 shows typical reformatted sagittal images of three selected cases using this technique. Five sagittal alignment parameters were measured from reconstructed sagittal CT images in accordance with the Spinal Deformity Study Group’s *Radiographic Measurement Manual*[44] for the entire group of 30 patients including: (i) proximal thoracic kyphosis (T2 to T5); (ii) mid/lower thoracic kyphosis (T5 to T12); (iii) global thoracic kyphosis (T2 to T12); (iv) thoracolumbar alignment (T10 to L2); (v) lumbar sagittal alignment (T12 to S1 lordosis). An additional two sagittal alignment measures were made; (vi) sagittal alignment of the instrumented levels, and (vii) sagittal alignment within the motion segment immediately distal to the instrumentation. 

The study group was also divided into subgroups based on the T5-12 Cobb angle on the standing radiograph before surgery. The patients were classified into hypokyphotic (HK,< 20°), normal (NK, 20-40°) or hyperkyphotic (>40°) groups based on the most commonly reported range of normal thoracic kyphosis 20-40° in the literature [17,45-50]. Each subgroup was analysed in terms of the sagittal alignment parameters listed above. Analysis was also performed to see if there was any significant difference in the correction of the patients who had a 4.5 versus the 5.5 mm rod.

Two blinded independent observers (Spinal Orthopaedic Surgeon, experienced Senior Research Assistant) measured the reformatted sagittal plane CT images using the ImageJ software on both the preoperative and 24 month postoperative CT scans of each patient. The observers were blinded to patient identity and the patient order was randomised with no pre-marking of vertebral endplates. To analyse the effect of supine versus standing posture on sagittal values, Cobb angles were measured before surgery and at 24 months after surgery on the sagittal radiographs, where endplate clarity was sufficient, and compared to the same measurements from the reformatted sagittal CT images.

#### Statistical analysis

Statistical analyses of the changes of the various sagittal parameters after surgery compared to before were performed using two-tailed paired Student’s t-tests. The paired t-tests were performed for the overall patient group, as well as for separate subgroups of patients (HK or NK and 4.5 or 5.5 mm rod). Comparison of supine CT and standing x-ray sagittal plane Cobb angles was performed using t-tests and least squares linear regression in SPSS (version 15.0, IBM, Armonk, NY).

The inter-observer variability for measurement of sagittal Cobb angles on reformatted sagittal CT images was assessed using the approach described by Bland and Altman [51,52]. The inter-observer difference (α) was calculated as;

(1) $$\Delta \alpha =\left|\alpha_{n}-\alpha_{m}\right|$$

where *n* and *m* are the Cobb angle measurements by the two observers. The 95% confidence intervals for inter-observer variability were calculated as 2.04 × *SD*_*inter*_ where *SD*_*inter*_ is the standard deviation of the inter-observer differences for all patients in the group (2.04 coefficient was determined using a *t*-distribution with 30 *dof*).

## Results

27 females and 3 males consented to participate in the study. The mean age at the time of surgery was 15.4 ± 3.7 years (range 9.9-27.8). All 30 patients had right sided major thoracic Lenke 1 Type curves with 19 patients further classified as lumbar spine modifier A, 8 as lumbar modifier B, and 3 with lumbar modifier C. The mean major thoracic Cobb angle for the group before surgery was 51.3 ±  7.1 (range 40–66) and decreased to mean 21.9 ±  8.2 (range 8–33) on the fulcrum bending radiographs. The mean secondary lumbar Cobb angle before surgery measured 31.6 ±  9.0 (range 15–50) and decreased to mean 7.9 ±  7.6 (range 0–28) on active side bending radiographs. Mean T5-T12 kyphosis Cobb angle before surgery on radiographs was 15.6° ± 9.6 (range −8 - 28) such that 17 patients were classified as exhibiting a hypokyphosis, 13 were normokyphotic (Table 1) and of note the largest T5-12 kyphosis was 28°.

**Table 1:** Number of patients with each curve type before surgery using the Lenke Classification and grouped according to the T5-T12 Cobb angle on standing radiographs before surgery

**T5-T12 kyphosis Cobb angle**	**<20°**	**20 to 40°**	**>40° (hyper)**	**Total**
	**(hypo)**	**(normal)**		
Lenke Type IA	12	7	0	**19**
Lenke Type IB	4	4	0	**8**
Lenke Type IC	1	2	0	**3**
**Total**	**17**	**13**	**0**	**30**

The upper level chosen was T5 in 13 cases, T6 in 15 cases and T7 in 2 cases. The lowest instrumented level was L1 in 3 cases, T12 in 20 cases, T11 in 5 cases and T10 in 2 cases. The mean number of levels fused and instrumented was 7.2 ± 0.7 (range 6–8). The postoperative low dose CT was performed at mean 2.2 ± 0.7 years (range 1.8-5.9) after surgery. The mean T5-T12 kyphosis Cobb angle at minimum 2 years after surgery was 27.4° ± 9.0 (range 8–45) showing a mean increase of 11.8° based on the standing sagittal radiographs. There was a single patient that remained by definition hypokyphotic on the 24 month follow-up radiograph (from 8° thoracic lordosis before surgery to 8° thoracic kyphosis after surgery).

Table 2 shows changes in sagittal alignment measures on CT two years after surgery for all patients compared to those before surgery. With the exception of thoracolumbar alignment (T10-L2) and sagittal alignment of the motion segment distal to the instrumentation, all changes as a result of surgery were statistically significant. For direct comparison, Table 2 also shows all possible sagittal Cobb angles as measured on standing radiographs (marked ‘x-ray’) for the same group of patients and indicates statistically significant differences between CT and X-Ray values.

**Table 2:** Mean sagittal parameters measured on supine low dose CT scans before surgery (degrees ± standard deviation) and at 2 years after surgery for 30 patients who underwent TASF for progressive scoliosis (note: positive angles represent kyphosis and negative angles represent lordosis)

**Sagittal Cobb angle (°)**		**Before surgery**	**2 years after surgery**	**Difference (°)**	**Significance**
**T5 - T12**	**CT**	10.3 ± 7.5	22.8 ± 9.8	+12.5	p < 0.001
	**(X-Ray)**	*15.6 ± 9.6	*27.4 ± 9.0	+11.8	p < 0.001
**T2 - T12**	**CT**	18.5 ± 8.7	26.9 ± 9.9	+8.4	p < 0.001
**Instrumented**	**CT**	8.3 ± 6.8	22.5 ± 10.3	+14.2	p < 0.001
**Levels**	**(X-Ray)**	*11.6 ± 7.5	23.6 ± 8.7	+12.0	p < 0.001
**T2 - T5**	**CT**	8.1 ± 5.4	3.9 ± 5.0	−4.2	p < 0.001
**T10 - L2**	**CT**	1.5 ± 6.8	3.4 ± 7.6	+1.9	p = 0.16
	**(X-Ray)**	−2.8 ± 7.1	2.9 ± 7.0	+5.7	p < 0.001
**Distal to Rod**	**CT**	2.1 ± 5.0	2.7 ± 4.6	+0.6	p = 0.57
	**(X-Ray)**	0.5 ± 3.6	1.5 ± 3.4	+1.0	p = 0.20
**T12 - S1**	**CT**	−51.1 ± 8.4	−57.3 ± 10.8	+6.2	p < 0.001
	**(X-Ray)**	*-56.2 ± 9.5	*-62.1 ± 9.5	+5.9	p < 0.001

For comparison with the CT measurements, sagittal parameters measured from standing radiographs are also shown (T2 was unable to be visualised on radiographs). *indicates statistically significant difference between CT and X-Ray values (paired t-test, p < 0.05).

**Figure 4:**
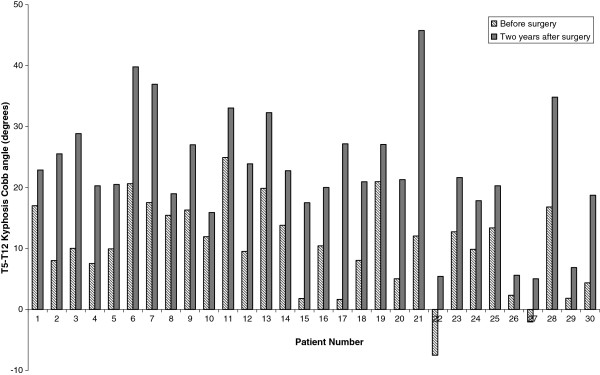
**Changes in T5-T12 kyphosis Cobb angle before and two years after surgery for all patients in the study measured from ethically approved low dose CT for research purposes**.

Figure 4 shows T5-T12 kyphosis before and after surgery measured on CT for each individual patient in the study. Table 3 gives CT sagittal alignment results for the hypokyphotic and normokyphotic subgroups before and after surgery and indicates statistically significant changes as a result of the surgical correction for each subgroup. The changes of the various sagittal parameters of the NK and HK groups behaved similarly to the sagittal changes of all 30 patients with regards to statistical significance. Table 4 shows the changes of all the sagittal parameters for the 4.5 and 5.5 mm rod groups and found there were no statistically significant differences between these groups of patients. Table 5 gives the inter-observer measurement variability (mean, standard deviation and 95% confidence intervals) for measurements of T5-T12, T2-T12, T10-L2, and T12-S1 sagittal Cobb angles by the two observers.

**Table 3:** Mean sagittal parameters measured on supine low dose CT scans before surgery and at 2 years after surgery for subgroups of patients who were classified preop as being (i) hypo-kyphotic (HK, n = 17) or (ii) Normo-kyphotic (NK, n = 13)

**Sagittal Cobb angle (°)**	**Before Surgery (± sd)**		**2 years postop (± sd)**		**Difference (°)**	
	**Hypo**	**Norm**	**Hypo**	**Norm**	**Hypo (−)**	**Norm (N)**
**T5 - T12**	6.6 ± 7.3	15.5 ± 3.7	18.6 ± 8.5	28.3 ± 8.5	+12.0*	+12.8*
**T2 - T12**	13.4 ± 6.2	25.2 ± 6.6	22.5 ± 8.4	32.2 ± 8.28	+ 9.1*	+ 7.0*
**Instrumented Levels**	5.3 ± 7.8	11.9 ± 3.8	18.5 ± 9.1	27.2 ± 8.5	+13.2*	+15.3*
**T2 - T5**	6.8 ± 5.1	9.7 ± 5.5	4.0 ± 5.4	3.9 ± 4.8	- 2.8*	- 5.8*
**T10 - L2**	0.7 ± 8.4	3.0 ± 5.5	2.6 ± 8.1	4.0 ± 7.2	+ 1.9	+ 1.0
**Distal to Rod**	1.3 ± 3.9	3.1 ± 6.2	2.1 ± 3.7	3.4 ± 5.7	+ 0.8	+ 0.3
**T12 - S1**	−49.5 ± 8.4	−53.3 ± 7.4	−55.0 ± 11.0	−61.9 ± 10.6	+ 5.5*	+ 8.6*

* indicates statistically significant difference between pre-operative and 2 year post-operative kyphosis measures (paired t-test, P < 0.05). No statistically significant differences were found between the HK and NK groups in terms of pre to postop changes for any of the sagittal Cobb measures (unpaired t-test, p < 0.05).

**Table 4:** Mean sagittal parameters measured on supine low dose CT scans before surgery and at 2 years after surgery for subgroups of patients who had either a (i) 4.5 mm rod (n = 14) or (ii) 5.5 mm rod (n = 16)

**Sagittal Cobb angle**	**Difference after Surgery (°)**	
	4.5 mm Rod	5.5 mm Rod
**T5 - T12**	13.5*	11.2*
**T2 - T12**	9.9*	6.7*
**Instrumented Levels**	16.4*	12.1*
**T2 - T5**	−3.6*	−4.5*
**T10 - L2**	2.2	0.9
**Distal to Rod**	1.2	0.0
**T12 - S1**	7.2*	6.6*

* indicates statistically significant difference between pre-operative and 2 year post-operative measures (paired t-test, P < 0.05). No statistically significant differences were found between the 4.5 and 5.5 mm rod groups in terms of pre to post op sagittal changes (unpaired t-test, p < 0.05).

**Table 5:** Inter-observer variability (in degrees) for sagittal Cobb angle measurement on supine low dose CT scans

	**T5-T12**		**T2-T12**		**T10-L2**		**T12-S1**	
**Inter-observer difference**	**Before surgery**	**After surgery**	**Before surgery**	**After surgery**	**Before surgery**	**After surgery**	**Before surgery**	**After surgery**
**Mean**	0.26	0.25	0.21	0.10	0.20	0.30	0.14	1.71
**SD**	3.04	2.34	3.67	2.77	3.10	2.95	3.88	3.77
**95% CI**	6.20	4.77	7.48	5.65	6.32	6.01	7.91	7.69

Table 6 compares the results of the current study with previous studies reporting changes in sagittal parameters on standing radiographs before and at a minimum two years after selective thoracic fusion surgery using anterior or posterior approaches.

**Table 6:** Previous publications on selective thoracic fusion surgery reporting changes in mean sagittal Cobb angles (in degrees) before surgery to minimum 2 years after surgery, as measured on standing sagittal radiographs

**Selective Thoracic Fusion Surgery**	**T5-T12**	**T2-T12**	**T10-L2**	**T12-S1**
**Anterior approach**				
Current Study (TASF, n = 30)				
Supine CT	+12.5	+8.4	+1.9	+6.2
Standing radiograph	+11.8		+5.7	+5.9
Betz et al., 1999 [17]	+16			+6
OASR (flexible rod), n = 78				
Rhee et al., 2002 [14]	+4		+1	+1
OASR, n = 23				
Wong et al., 2004 [34]		+7		−1
TASF, n = 12				
Potter et al., 2005 [7]		+5.7		−1.4
OASR, n = 20				
Newton et al., 2005 [29]	+10			
TASF, n = 45				
Sucato et al., 2008 [5]	+6.2		+1.1	+8.6
OASR (n = 93) & TASF (n = 42) combined				
Newton et al., 2008 [35]	+10.1		−0.2	+6.8
TASF, n = 25				
Yoon et al., 2008 [36]				
TASF 4 mm stainless steel rod, n = 24	+9.5			+4
TASF 4.75 mm titanium alloy, n = 25	+6.5			−1
Lonner et al., 2009 [18]	+8.7	+6.6		+5.2
TASF, n = 26				
Lonner et al., 2009 [4]	+4.3			+4.6
TASF, n = 17				
Hay et al., 2009 [8]	+12.3			
TASF, n = 106				
Tis et al., 2009 [19]	+8.0			
OASR, n = 85				
Newton et al., 2010 [16]	+7.9			+3.0
TASF (n = 71) & OASR (n = 97) combined				
**Posterior approach**				
Betz et al., 1999 [17]	+1			+2
Post Open (segmental hooks/rods), n = 100				
Rhee et al., 2002 [14]	−2		+1	+2
Post hybrid (screws/hooks/wires), n = 40				
Wong et al., 2004 [34]		−5		+2
Post Open (segmental hooks/rods), n = 19				
Suk et al., 2005 [53]	+2.5			−1.0
PPS, n = 151 (King II and III)				
Potter et al., 2005 [7]		−4.4		−7.4
PPS, n = 20				
Vora et al., 2007 [20]				
*Note – Kyphosis levels not defined	−12.0			
Post hooks, wires, n = 24	+2.1			
Post hybrid (screws/hooks/wires), n = 23				
PPS, n = 25	−10.9			
Sucato et al., 2008 [5]				
Post hybrid (screws/hooks/wires), n = 86	+0.4		+1.7	+4.4
Post Open (hooks only), n = 132	+1.9		+4.5	−1.8
Lehman et al., 2008 [21]	−9.9		−4.6	−2.9
PPS, n = 114				
Lonner et al., 2009 [4]	+1.6			+3.4
PPS, n = 17				
Quan et al., 2010 [22]	−8.4			
PPS, n = 49				
Newton et al., 2010 [16]	−2.6			−5.6
PPS, n = 83				
Abel et al., 2011 [54]	+0.9	+3.4	+5.0	−0.5
Post Open (pedicle screws, hybrid), n = 123				

TASF (thoracoscopic anterior spinal fusion with single rod, OASR (open anterior single rod), PPS (posterior pedicle screws).

**Figure 5:**
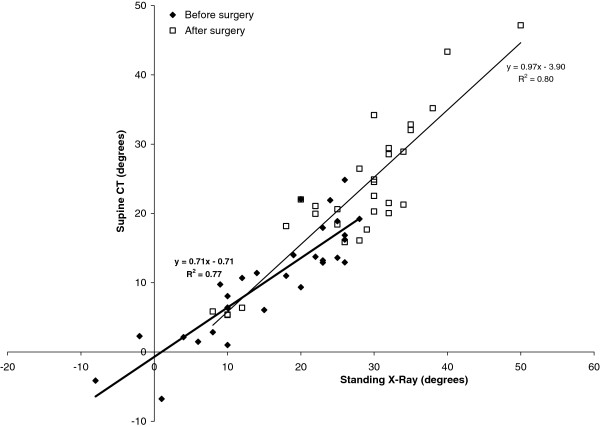
**Comparison of T5-T12 kyphosis between supine CT and standing radiographs both before and two years after surgery.** Linear regression lines and equations are given (bold = before surgery).

**Figure 6:**
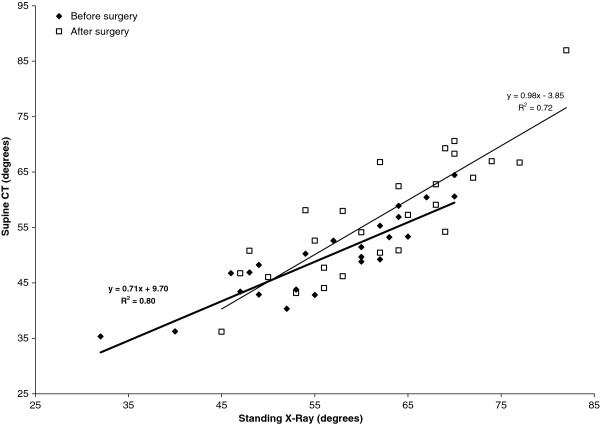
**Comparison of T12-S1 lordosis between supine CT and standing radiographs both before and two years after surgery.** Linear regression lines and equations are given (bold = before surgery).

Figures 5 and 6 present a comparison of supine CT versus standing x-ray Cobb angles for thoracic kyphosis (Figure 5) and lumbar lordosis (Figure 6) respectively, both before and two years after surgery. Linear regression equations are shown on the graph for each best fit line both before and after surgery in the form *y = mx + c*, where *x* is the sagittal plane Cobb angle measured on standing radiographs, and *y* is the Cobb angle measured on supine CT. These regression equations provide a useful means to convert between standing and supine sagittal profile measures. For example, a standing lumbar lordosis Cobb angle of 55º before surgery would be expected to reduce to 0.71 × 55° + 9.7° = 49° with the patient in a supine position. The standard errors of the slopes of the regression equations were 0.121 (T12-S1 lordosis post-op), 0.074 (T12-S1 lordosis pre-op), 0.093 (T5-T12 kyphosis post-op), and 0.071 (T5-T12 kyphosis pre-op), all of which were statistically significant at the P < 0.001 level.

## Discussion

Restoration of normal sagittal profile is an important goal of scoliosis correction surgery. The aim of this study was to provide a detailed analysis of sagittal profile correction following TASF, using both standing plane radiographs and supine low dose CT scans of the same patient group. CT was useful in addition to lateral radiographs due to the superior endplate clarity afforded by the reformatted CT images. The use of supine CT also potentially avoids the inherent variability in upright standing posture due to stance variations between subsequent sagittal radiographs [55]. However, the use of supine CT also raises questions about the applicability of the resulting sagittal profile measurements to clinically relevant standing postures, and in this study we provide a detailed comparison of standing versus supine sagittal profile measurements for the same patients. To our knowledge, no previous study has compared sagittal profile measurements before and after scoliosis surgery between standing radiographs and supine CT, nor reported on detailed changes in sagittal profile using the clearly defined vertebral endplate visualisation afforded by low dose CT. We also wished to compare the results of the current study with existing literature using plane radiographic measurements of sagittal profile following other selective thoracic fusion procedures (Table 6).

After selective thoracic fusion, the lumbar spine needs to adapt to the altered shape of the thoracic spine to maintain coronal and sagittal balance [16]. This spontaneous correction of the lumbar compensatory curve in the coronal plane has been evaluated for various surgical approaches with varying reports as to the superiority of correction between anterior and posterior approaches [6,7,17,20,56,57]. The post-operative response of the lumbar spine in the sagittal plane is thought to be a consequence of the change in thoracic kyphosis achieved during surgery. A number of recent studies [4,5,7,14,16] have found that anterior techniques for the correction of thoracic scoliosis are more kyphogenic than posterior approaches. Multiple discectomies and compression along the rod lead to shortening of the anterior column and immediate increases in the thoracic kyphosis at the first erect radiograph after surgery, with further increases reported two years after anterior selective thoracic fusion [5,7,8,14,16,18]. Prior studies by our group on the larger cohort have reported complications associated with this type of surgery [8,41,58]. In the current cohort of 30 patients, there were 3 rod fractures and 3 top screw pullouts found by the most recent follow-up. Note that as previously reported, rod fracture is associated with a minimal loss of correction and tended to occur in the earliest patients in the series with only 3 rod fractures found from the most recent 150 cases in the larger series.

A number of prior studies have noted the poor quality of sagittal radiographs with regard to the visualisation of vertebral endplates, especially in the mid and upper thoracic regions of the spine. For example, Dang *et al*[37] reported excellent intra-observer reproducibility for coronal plane radiograph measurements but for sagittal radiographs, examiners were found to have only fair to good reproducibility for angles measured from upper thoracic vertebrae, such as T2 or T5, and poor inter-observer agreement when measuring spinal levels below T9. Dang *et al’s* paper concluded that sagittal parameters measured on traditional radiographs do not provide valuable information because they cannot be measured reproducibly or reliably. The difficulties with measuring sagittal parameters on lateral radiographs [37-39] are avoided with reformatted sagittal CT reconstructions due to the superior endplate clarity afforded by this imaging modality. In the current study, the 95% confidence intervals for inter-observer variability of sagittal Cobb angle measurements (range 5-8°, Table 5) are comparable with previously published 95% confidence intervals for coronal Cobb angle measurement from supine CT scans [59]. This suggests that the use of CT allows equivalent clarity for either sagittal or coronal plane Cobb angle measurements.

This study confirms that TASF is a kyphosing technique which has a similar corrective effect on patients who are hypokyphotic or normokyphotic before surgery (Table 3). Those receiving a 4.5 mm rod had a slightly greater increase of their kyphosis across the instrumented segment than the group receiving the 5.5 mm rod which is in contrast to an earlier study using posterior approaches where the use of larger diameter titanium rods (6.35 vs 5.5 mm) resulted in larger thoracic kyphosis after surgery [50]. However, a recently published paper on 49 TASF cases [36] found similar results to the current study reporting a greater increase in kyphosis when using a smaller diameter rod (4.0 mm stainless steel in earlier patients vs. 4.75 mm titanium alloy) but rather than interpret the difference as being the result of the different implant types, suggested evolving surgeon experience in patient selection was the most likely factor influencing the different sagittal changes. The 4.5 mm rod group in the current study were also the earlier cases in our larger series undergoing TASF so may also have been affected by a similar patient selection issue, although our differences were not statistically significant. All 30 patients in the study had some increase in thoracic kyphosis following TASF surgery according to CT (Figure 4) and X-Ray measures, with 26 patients found to be in the normokyphotic range on the minimum 24 months after surgery radiographs. One patient was classified as being hyperkyphotic two years after surgery (T5-T12 kyphosis 45° on CT and X-Ray) and continues to be monitored six years later and to date has not required additional surgery. AIS is a triplanar deformity and in Lenke type 1 scoliosis, the results presented here suggest that anterior correction is capable of addressing the sagittal component of both the instrumented and adjacent non instrumented segments. The corrective forces exerted by single rod anterior constructs results in a flexion moment which increases the kyphosis across the instrumented levels. The un-instrumented lumbar spine must in turn balance the kyphotic curve above so any increase in thoracic kyphosis will see a corresponding increase in the lumbar lordosis of the patient. This is evidenced in the current study (Table 2) where T2-T12 kyphosis increased by a mean 8.4° and the T12-S1 lordosis increased by mean 6.2°.

Table 6 compares the results of the current study with previous studies reporting changes in sagittal contour after scoliosis correction surgery. This table shows that posterior approaches either exacerbate the existing thoracic hypokyphosis (at worst 12 degrees [20]), or only achieve small increases in kyphosis in the order of 1-2°. By contrast, anterior thoracic fusion procedures consistently increase T5-T12 kyphosis by between 4 - 12° at two years after surgery. With respect to lumbar lordosis, Table 6 reports lumbar lordosis flattening as much as 7.4° following posterior selective thoracic procedures, whereas again by contrast anterior approaches report a deepening of the lumbar lordosis by as much as 8.6° in response to the kyphosing surgical effect in the thoracic spine. The results of the meta-survey of prior studies in Table 6 suggest that anterior correction is more capable of addressing the sagittal component of both the instrumented and adjacent non instrumented segments for AIS patients.

Use of the supine position for CT-based sagittal profile measurement clearly changes the geometry of the spine relative to the standing posture, but the comparative results in this study (Table 2, Figures 5, 6) show that there is a predictable (linear) relationship between supine and standing sagittal profile measurements. Of note is that mean kyphosis across the instrumented levels after surgery changed minimally between supine and standing, whereas the uninstrumented lumbar lordosis and T5-T12 kyphosis each demonstrated significant differences (mean 4.8°) due to the change of posture. It is not being suggested that CT scans should replace standing radiographs for scoliosis assessment, but for the group of patients examined here for research purposes, the paired CT data before and after surgery uniquely provided a superior imaging modality (in terms of image contrast and endplate clarity) for analysing the effects of TASF on sagittal plane deformity. There are both advantages and disadvantages to supine measurement of sagittal profile. Use of the supine position provides an ‘unloaded’ configuration of the spine which is not subject to variations in standing posture due to arm positioning [46,55,60], time of day [61], or muscle activation strategy [62,63], all of which can affect sagittal Cobb measurements. Further, relative rotation between the pelvis and ribcage can vary between subsequent standing radiographs whereas the supine position standardises many of these variables. Supine measurements are also valuable in biomechanical modelling of scoliosis, since the supine position provides an approximate zero load configuration for the spine which can be used as a starting point for biomechanical simulations. A disadvantage of supine imaging and a limitation of this study is that sagittal balance and the role of pelvic incidence in the standing position cannot be assessed. Further, the standing position is relevant to a condition such as scoliosis where gravity is known to affect the magnitude of the deformity. Recent advances in multi-slice CT are allowing lower radiation doses and faster acquisition times which will make CT an increasingly useful research tool for three-dimensional biomechanical studies of scoliosis correction. Also, low dose standing biplanar systems (such as EOS) are expected to play an important future role in scoliosis imaging and surgical planning.

Thoracoscopic anterior instrumented fusion significantly improves global thoracic kyphosis (T2-T12), thoracic kyphosis (T5-T12), lumbar lordosis (T12-S1) and instrumented segment kyphosis while simultaneously correcting and stabilising the coronal and rotational plane deformities. The results of this study show that the technique reliably increases thoracic kyphosis and lumbar lordosis while preserving proximal and distal junctional alignment in thoracic AIS patients.

## Competing interests

The authors declare they have no competing interests.

## Authors’ contributions

GNA and RDL participated in the conception and design of the study, performed the surgical procedures, and were involved in the drafting and reviewing of the manuscript. EV performed angle measurements for the study, and participated in the literature review and drafting of the initial manuscript. CJA and MTI participated in the design of the study, the literature review, produced the radiographic and CT images for analysis, performed the data analysis and statistical analysis, drafting and reviewing of the manuscript, the submission and revisions to the final manuscript. All authors read and approved the final manuscript.
